# An algorithm for space–time block code classification using higher-order statistics (HOS)

**DOI:** 10.1186/s40064-016-2139-z

**Published:** 2016-04-26

**Authors:** Wenjun Yan, Limin Zhang, Qing Ling

**Affiliations:** Institute of Information Fusion, Naval Aeronautical and Astronautical University, Erma Road, Zhifu District, Yantai, 264001 China

**Keywords:** Space–time block code (STBC), Signal classification, Higher-order statistics (HOS), Single receive antenna

## Abstract

This paper proposes a novel algorithm for space–time block code classification, when a single antenna is employed at the receiver. The algorithm exploits the discriminating features provided by the higher-order cumulants of the received signal. It does not require estimation of channel and information of the noise. Computer simulations are conducted to evaluate the performance of the proposed algorithm. The results show the performance of the algorithm is good.

## Background

Blind signal classification of communication signals plays a pivotal role in both civilian and military applications, such as electronic warfare, radio surveillance, civilian spectrum monitoring, and cognitive radio systems (Axell et al. 2012; Dobre 2015; Dobre et al. 2005, 2007). The research on signal classification for multiple input multiple output (MIMO) scenarios is at an incipient stage. Regarding space–time block code (STBC) classification algorithms, they can be divided into several general categories: likelihood-based (Choqueuse et al. 2010), subspace-based (Swindlehurst and Leus 2002; Zhao et al. 2014), second-order statistics based (Via and Santamaria 2008a, b), cyclostationarity based (DeYoung et al. 2008; Shi et al. 2007; Marey et al. 2012), higher-order based (Choqueuse et al. 2008a, b, 2011; Eldemerdash et al. 2013a, b) and correlation function based (Marey et al. 2014; Mohammadkarimi and Dobre 2014).

The likelihood-based algorithm evaluate the likelihood function of the received signal ,and employ the maximum likelihood criterion for decision making. However, the likelihood-based algorithm need channel estimation and signal imformation (Choqueuse et al. 2010). To avoid the drawbacks of likelihood-based algorithm, several authors have investigated the use of subspace (Swindlehurst and Leus 2002; Zhao et al. 2014) and second-order statistics (SOS) (Via and Santamaria 2008a, b) algorithm. However excluding some specific low-rate codes, these approaches fail to extract the channel in a full-blind contex (Swindlehurst and Leus 2002; Zhao et al. 2014; Via and Santamaria 2008a, b). These semi-blind methods cannot be employed in a non-cooperative scenario since they require modification of the transmitter. To avoid the drawbacks above, the cyclostationarity-based and higher-order based algorithms are proposed. Most of the paper formed with more than a single antenna (Choqueuse et al. 2008a, b; Eldemerdash et al. 2013b; Choqueuse et al. 2011). Some of the articles study the classification of spatial multiplexing (SM) and Alamouti STBC (DeYoung et al. 2008; Shi et al. 2007; Eldemerdash et al. 2013b), and others study a large pool (Marey et al. 2012; Choqueuse et al. 2008a, b, 2011). Literature (Marey et al. 2014) and literature (Mohammadkarimi and Dobre 2014) performe under frequency-selective channels and impulsive noise respectively . However, few articles illustrate the STBC classification when a single antenna is employed at the receiver (Eldemerdash et al. 2013a; Mohammadkarimi and Dobre 2014). Since in reality the requirement cannot always be met, blind classification for STBC are of interest when a single receive antenna is available.

This paper proposed an efficient algorithm based on Higher-order cumulants for classification of STBC. We use the properties of higher-order cumulants to avoid the effect of noise. We exploit features based on fourth-order cumulants, and divide the STBCs with an interval detector. The proposed algorithm performs well in the simulation and does not need channel estimation and signal information.

## Signal model and assumption

### Signal model

We consider a wireless communication system which employs linear space–time block coding with multiple transmit antennas. Each symbol is encoded to generate $n_t$ parallel signal sequences of length *L*. The sequences are transmitted simultaneously with $n_t$ antennas in *L* consecutive time periods. The *k*th $n_t\times L$ matrix can be denoted by $C(S_k)$, from a block of *n* symbols denoted $s=[s_1,s_2,\ldots ,s_n]^T$.

The received signal is assumed to be encoded by one of the following STBCs^1^:SM (Choqueuse et al. 2008a) with $n_t=1$ and *L* = 1, Alamouti STBC (Al for short) code (Alamouti 1998) with $n_t=2$ and *L* = 2 (orthogonal with rate 1), ST3 (Choqueuse et al. 2008a) with $n_t=3$ and *L* = 8 (orthogonal with rate $\frac{3}{4}$), ST4 (Tarokh et al. 1999) with $n_t=4$ and *L* = 8 (orthogonal with rate $\frac{3}{4}$).

The matrix of each STBC is defined as

1 $$C^{SM}(S)=s_j,\quad j=1,2,3,\ldots$$

2 $$C^{Al}(S)= \left[ \begin{array}{ll} s_1 &\quad -s_2^* \\ s_2 &\quad s_1^* \end{array} \right]$$

3 $$C^{ST3}(S)= \left[ \begin{array}{cccccccc} s_1 &\quad -s_2 &\quad -s_3 &\quad -s_4 &\quad s_1^* &\quad -s_2^* &\quad -s_3^* &\quad -s_4^* \\ s_2 &\quad s_1 &\quad s_4 &\quad -s_3 &\quad s_2^* &\quad s_1^* &\quad s_4^* &\quad -s_3^* \\ s_3 &\quad -s_4 &\quad s_1 &\quad s_2 &\quad s_3^* &\quad -s_4^* &\quad s_1^* &\quad s_2^* \\ \end{array} \right]$$

4 $$C^{ST4}(S)= \left[ \begin{array}{cccccccc} s_1 &\quad -s_2 &\quad -s_3 &\quad -s_4 &\quad s_1^* &\quad -s_2^* &\quad -s_3^* &\quad -s_4^* \\ s_2 &\quad s_1 &\quad s_4 &\quad -s_3 &\quad s_2^* &\quad s_1^* &\quad s_4^* &\quad -s_3^* \\ s_3 &\quad -s_4 &\quad s_1 &\quad s_2 &\quad s_3^* &\quad -s_4^* &\quad s_1^* &\quad s_2^* \\ s_4 &\quad s_3 &\quad -s_2 &\quad s_1 &\quad s_4^* &\quad s_3^* &\quad -s_2^* &\quad s_1^* \\ \end{array} \right]$$

We consider a receiver with a single antenna, and assume that the length and time alignment of the STBC blocks are unknown. Without loss of generality, we assume the first received symbol ,denoted by $Y_0$, intercepts the ($k_1+1$)th column, $1\le k_1 {<}L$, of the *b*th transmitted block, denoted by $G_{k_1}(X_b)$. Under these assumptions, the *k*th received symbol, $Y_k$ can be described as (Choqueuse et al. 2008a)

5 $$Y_k=HC(S_k)+B_k$$

where $C(S_k)=G_{p}(X_q)$, with $p=(k+k_1)$ mod *L*, $q=b+(K+k_1)$ div *L*, and *z* mod *L* and *z* div *L* denoting respectively the remainder and the quotient of the division *z*/*L*. $H=[h_1,\ldots ,h_{n_t}]$ denotes the vector of the fading channel coefficients, which are considered to be constant over the observation period. $B_k$ represents the complex additive white Gaussian noise (AWGN).

### Main assumptions

In this study, the following conditions are assumed to hold.

(AS1) The data symbols are assumed to belong to an *M*-PSK or *M*-QAM signal constellation , and consist of independent and identically distributed random variables with zero mean and $E[|s|^2]=E[|s|^4]=1$, $E[s^2]=E[(s^*)^2]=0$, and $E[s^4]=E[(s^*)^4]=-1$ Eldemerdash et al. (2013b).

(AS2) The received signal is affected by a frenquency-flat Nakagami-*m* fading channel Beaulieu and Cheng (2005), with *m* = 3, and $E[|h_i|^2]=E[|h_i|^4]=1$, $E[h_i^2]=i$, and $E[h_i^4]=-1$, where $i=1,\ldots ,n_t$.

(AS3) The noise vector $B_k$ is a complex stationary, and ergodic Gaussian vector process, independent of the signals, with zero mean and variance $\sigma ^2$. It implies that: $E[B_kB_k^H]=\sigma ^2L$. The SNR is defined as $10\log _{10}{\left( \frac{n_t}{\sigma ^2}\right) }$ (Swami and Sadler 2000).

AS4) The received signal intercepts a whole number $N_b$ of space–time blocks $Y=[Y_1,\ldots ,Y_{N_b}]$, i.e., the first and last intercepted samples correspond to the start and the end of a space–time block, respectively.

## Classification based on HOS

In this section, we exploit the feature by using Higher-order cumulants. We will first define the fourth-order cumulants which we propose to use, discuss how they can be estimated from the data, and then give the theoretical values for various STBCs.

### Definitions

For a complex-valued stationary random process *y*(*n*), second-order moments can be defined in two different ways depending on placement of conjugation (Swami and Sadler 2000)

6 $$\begin{aligned} C_{20}&=E\left[ y(n)^2\right] \\ C_{21}&=E\left[ y(n)y(n)^*\right] =E\left[ |y(n)|^2\right] \\ \end{aligned}$$

Fourth-order moments and cumulants can be written in two way^2^

7 $$\begin{aligned} C_{40}&=cum(y(n),y(n),y(n),y(n)) \\ C_{42}&=cum(y(n),y(n),y^*(n),y^*(n)) \\ \end{aligned}$$

The statistics in Eqs. (6) and (7) are the zeroth lags of the correlations and fourth-order of *y*(*n*). For zero-mean random variable *w*, *x*, *y*, and *z*, the fourth-order cumulants can be written as

8 $$\begin{aligned} cum(w,x,y,z)&=E(wxyz)-E(wx)E(yz)\\&\quad -E(wy)E(xz)-E(wz)E(xy) \end{aligned}$$

### Sample estimates

We assume the complex-valued stationary random process *y*(*n*) is zero-mean. In practice, the sample mean is removed before cumulants estimation. The correlations of *N* samples are given by

9 $$\begin{aligned} \hat{C}_{20}&=\frac{1}{N}\sum _{n=1}^Ny^2(n) \\ \hat{C}_{21}&=\frac{1}{N}\sum _{n=1}^N|y(n)|^2 \end{aligned}$$

Where the superscript $\hat{\;}$ denote a sample average. The fourth-order cumulants can be written as

10 $$\begin{aligned} \hat{C}_{40}&=\frac{1}{N}\sum _{n=1}^N{y^4(n)-3\hat{C}_{20}^2} \\ \hat{C}_{42}&=\frac{1}{N}\sum _{n=1}^N{|y(n)|^4-|\hat{C}_{20}|^2-2\hat{C}_{21}^2} \end{aligned}$$

In particular, the higher-order (higher than 2nd) cumulants of zero-mean Gaussian symbols is zero. We assume the cumulants of noise is $\hat{C}_{xy,g}$, then $\hat{C}_{xy,g}=0(x{>}2)$. The analysis of higher-order statistic of received symbol is actually analysis of non-Gaussian signal. When evaluate the fourth-order cumulants of received signal, we can ignore the effect of AWGN (Zhang 2000). The fourth-order cumulants of received signal can be write as

11 $$\hat{C}_{4x,y}=\hat{C}_{4x,HC(s)}+\hat{C}_{4x,g}\approx \hat{C}_{4x,HC(s)}$$

where, $\hat{C}_{4x,y}$ represents the sample estimate of the fourth-order cumulants of received signal, $\hat{C}_{4x,HC(S)}$ represents the sample estimate of the fourth-order cumulants of received signal without noise, $\hat{C}_{4x,g}$ represents the estimate of fourth-order cumulants of noise.

### Theoretical values

Here, we consider the theoretical values of the fourth-order cumulants in Eq. (7) for various STBCs. The theoretical values are obtained by computing the ensemble averages over the ideal noiseless transmitted signal. We define $X^{STBC}=HC^{STBC}(S)$ as the noiseless transmitted signal, where $H^{SM}$, $H^{Al}$, $H^{ST3}$, $H^{ST4}$ can be expressed by $[h_0]$, $[h_0 \, h_1]$, $[h_0 \, h_1 \, h_2]$, $[h_0 \, h_1 \, h_2 \, h_3]$ respectively, $C^{STBC}$ is corresponding to Eqs. (1)–(4). The fourth-order cumulants for various STBCs are respectively defined as

12 $$\begin{aligned} C_{40}^{SM}&=E\left( X^{SM}X^{SM}X^{SM}X^{SM}\right) -3E\left( X^{SM}X^{SM}\right) \\&=E\left( h_0^4s_1^4-3h_0^2s_1^2\right) =E\left( h^4s^4-3h^2s^2\right) =1 \end{aligned}$$

13 $$\begin{aligned} C_{42}^{SM}&=E\left( X^{SM}X^{SM}{X^{SM}}^*{X^{SM}}^*\right) -E\left( X^{SM}X^{SM}\right) E\left( {X^{SM}}^*{X^{SM}}^*\right) \\&\quad -2E^2\left( X^{SM}{X^{SM}}^*\right) \\&=E\left( h_0^2s_1^2{h_0^*}^2{s_1^*}^2\right) -E\left( h_0^2s_1^2\right) E\left( {h_0^*}^2{s_1^*}^2\right) -2E\left( h_0s_1{h_0^*}{s_1^*}\right) ^2\\&=E\left( |h_0|^4|s_1|^4\right) -E\left( h_0^2s_1^2\right) E\left( {h_0^*}^2{s_1^*}^2\right) -2E\left( |h_0|^2|s_1|^2\right) ^2\\&=-1 \end{aligned}$$

14 $$\begin{aligned} C_{40}^{Al}&=E\left( X^{Al}X^{Al}X^{Al}X^{Al}\right) -3E\left( X^{Al}X^{Al}\right) \\&=\frac{1}{2}E\left[ \left( h_0s_1+h_1s_2\right) ^4+\left( -h_0s_2^*+h_1s_1^*\right) ^4\right. \\&\quad \left. -3\left( h_0s_1+h_1s_2\right) ^2-3\left( -h_0s_2^*+h_1s_1^*\right) ^2\right] \\&=\frac{1}{2}E\left[ h_0^4s_1^4+h_1^4s_2^4+6h_0^2h_1^2s_1^2s_2^2+h_0^4\left( s_2^*\right) ^4+h_1^4\left( s_1^*\right) ^4\right. \\&\quad \left. +6h_0^2h_1^2\left( s_1^*\right) ^2\left( s_2^*\right) ^2-3h_0^2s_1^2-3h_1^2s_2^2-3h_0^2\left( s_2^*\right) ^2-3h_1^2\left( s_1^*\right) ^2\right] \\&=2E\left( h^4s^4\right) \\&=2 \end{aligned}$$

15 $$\begin{aligned} C_{42}^{Al}&=E\left( X^{Al}X^{Al}{X^{Al}}^*{X^{Al}}^*\right) -E\left( X^{Al}X^{Al}\right) E\left( {X^{Al}}^*{X^{Al}}^*\right) -2E^2\left( X^{Al}{X^{Al}}^*\right) \\&=\frac{1}{2}\left\{ E\left[ \left( h_0s_1 + h_1s_2\right) ^2\left( h_0^*s_1^* + h_1^*s_2^*\right) ^2\right] - E\left[ \left( h_0s_1 + h_1s_2\right) ^2\right] E\left[ \left( h_0^*s_1^* + h_1^*s_2^*\right) ^2\right] \right. \\&\quad -2E\left[ \left( h_0s_1 + h_1s_2\right) \left( h_0^*s_1^* + h_1^*s_2^*\right) \right] ^2 + E\left[ \left( -h_0s_2^* + h_1s_1^*\right) ^2\left( -h_0^*s_2 + h_1^*s_1\right) ^2\right] \\&\quad -E\left[ \left( -h_0s_2^*+h_1s_1^*\right) ^2\right] E\left[ \left( -h_0^*s_2+h_1^*s_1\right) ^2\right] \\&\quad \left. -2E\left[ \left( -h_0s_2^*+h_1s_1^*\right) \left( -h_0^*s_2+h_1^*s_1\right) \right] ^2\right\} \\&=\frac{1}{2}\left[ E\left( |h_0|^4|s_1|^4 + |h_1|^4|s_2|^4 + |h_0|^4|s_2|^4 + |h_1|^4|s_1|^4 + 8|h_0|^2|h_1|^2|s_1|^2|s_2|^2\right) \right. \\&\quad \left. -2E\left( |h_0|^2|s_1|^2+|h_1|^2|s_2|^2\right) ^2-2E\left( |h_0|^2|s_2|^2+|h_1|^2|s_1|^2\right) ^2\right] \\&=-2 \end{aligned}$$

Since the derivation of the fourth-order cumulants for ST3 and ST4 is too long, we do not give the detailed derivation. They are similar to the derivation of SM and Al.

**Table 1:** Theoretical cumulants statistics *C*_40_ and *C*_42_ for various STBCs, and variances of their sample estimates

STBC	*C*_40_	*C*_42_	$Nvar(\hat{C}_{40})$			$Nvar(\hat{C}_{42})$		
			0 dB	5 dB	10 dB	0 dB	5 dB	10 dB
SM	1	−1	0.12	0.01	0.00	0.02	0.00	0.00
Al	2	−2	0.14	0.08	0.08	0.02	0.01	0.01
ST3	3	−3	0.81	0.71	0.41	0.10	0.08	0.05
ST4	4	−4	2.93	2.85	2.55	0.19	0.19	0.15

The theoretical values are described in Table 1. Column 2 shows *C*_40_ of STBCs, and column 3 shows *C*_42_. We can see that, The theoretical values of cumulants are different in various STBCs. The specific algorithm to be used depends upon the difference.

## Threshold analysis

In this section, we develop thresholds for the tests in the hierarchical classification scheme. In order to do this, we need to derive expressions for the variance of the sample estimates of the cumulants in Eq. (10). The variance expressions are estimated by 1000 Monte Carlo trail for each $\xi \in \{SM, Al, ST3, ST4\}$.

From Table 1, we note that the value of *C*_40_ or *C*_42_ and their sample variances are different for different STBCs. Consider a statistic *S*, Which is Gaussian with mean $\mu _i$ and variance $\sigma ^2_i$ under hypothesis $H_i$, *i* = 0, 1. Assume wlog that $\sigma _0^2 < \sigma _1^2$. Then the likelihood ratio test (LRT) for achieving minimum probability of error, assuming equal priors, is an interval detector, which can be written as Srinath et al. (1996)

16 $$decide \; H_0\; if\; S \in \; [\mu -a, \mu +a]$$

where

17 $$\mu \,{:}{=}\,\left( \frac{\mu _0}{\sigma ^2_0}-\frac{\mu _1}{\sigma _1^2}\right) \frac{\sigma _0^2\sigma _1^2}{\sigma _1^2-\sigma _0^2}$$

and

18 $$\alpha ^2:=\frac{\sigma _0^2\sigma _1^2}{\sigma _1^2-\sigma _0^2} \left[ \ln \frac{\sigma _1^2}{\sigma _0^2}+\frac{(\mu _1-\mu _0)^2}{\sigma _1^2-\sigma _0^2}\right]$$

if $\sigma _0^2=\sigma _1^2$, we have a threshold detector; thus, if $\mu _0{<}\mu _1$, we decide $H_0$ if $S{<}(\mu _0+\mu _1)/2$. From Table 1, we see that the variances of $\hat{C}_{40}$ and $\hat{C}_{42}$ are approximately the same for members of the STBCs, thus justifying the use of the threshold detector. The variances of $\hat{C}_{42}$ are more approximate to each other than $\hat{C}_{40}$, we tend to choose $\hat{C}_{42}$ as the threshold detector.

We define a four-class problem based on the STBCs given by

19 $$\Omega _4=\{SM,\, Al,\, ST3,\, ST4\}$$

For a given SNR, one can compute the optimal threshold under the assumption that $\hat{C}_{42}$ is Gaussian. Let $\mu _k$ and $\sigma ^2_k$ denote the mean and variance of the statistic, *S*, under the *k*th hypothesis; From Table 1, we see $\mu _1 {<} \mu _2< \mu _3 < \mu _4$. A simplifying approximation is to consider that the variances are all equal: in this case, the detection rule is to choose

20 $$H_k \, : \, \frac{\mu _{k-1}+\mu _k}{2}<S<\frac{\mu _k+\mu _{k+1}}{2}$$

with $\mu _0=\infty$ and $\mu _5=-\infty$, We assume that the variances is equal; thus, regardless of the actual noise variance, the decision rule can be written as Swami and Sadler (2000)

21 $$\begin{aligned}&\hat{C}_{42}>-1.5\, \Rightarrow \, SM \\ -1.5 \ge \,&\hat{C}_{42}>-2.5 \Rightarrow Al \\ -2.5 \ge \,&\hat{C}_{42}>-3.5 \Rightarrow ST3 \\ -3.5 \ge \,&\hat{C}_{42} \Rightarrow ST4 \end{aligned}$$

The average probability of correct classification is Eldemerdash et al. (2013b)

22 $$P_c=\frac{1}{4}\sum _{\xi \in \Omega _4}P(\xi |\xi )$$

One thousand of Monte Carlo trails is performed to calculate $P(\xi |\xi )$, where $\xi \in \Omega _4$.

## Simulation results

In this section, a variety of simulation experiments are presented illustrating the performance of the proposed classification schemes. For each Monte Carlo trial, the appropriate normalized statistics $\hat{C}_{42}$ is estimated via Eqs. (9) and (10), based on *N* data samples, and the additive noise is complex White Gaussian, via QPSK modulation. All results are based on 1000 Monte Carlo trials.

**Fig. 1:**
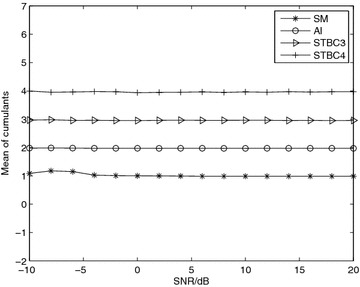
Mean of the fourth-order cumulants for different received signals with 1000 Monte Carlo trails, versus SNR and *N* = 8192 over Nakagami-*m* fading channel, *m* = 3

*Simulation 1* Mean of the fourth-order cumulants for different received signals. Figure 1 presents the mean of the fourth-order cumulants with respect to the different four STBCs over Nakagami-*m* fading channel with *m* = 3 at SNR = 10 dB. In order to observe the change of the cumulants of the samples and compare with the theoretical values, we give a large number of received samples. We decide the sample number *N* = 8192. In the figure, the ordinate represents the value of fourth-order cumulants of the four STBCs. We can see that under a large number of received samples, the value of fourth-order cumulants of STBCs tend to four different steady-state value corresponding to the theoretical values, $\{-1,-2,-3,-4\}$. The specific algorithm makes use of the character to classify different STBCs.

**Fig. 2:**
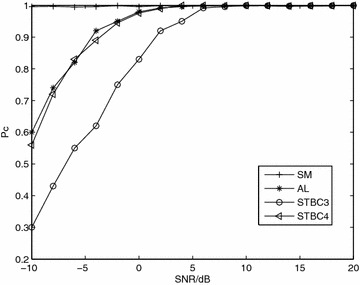
Probability of correct classification, *Pc*, versus SNR with QPSK modulation and *N* = 8192 over Nakagami-*m* fading channel, *m* = 3

*Simulation 2* Comparative performance of the four coding schemes. Figure 2 presents the performance of the four STBCs over Nakagami-*m* fading channel with *m* = 3. In the figure, the probability of correct classification for SM is almost 1 and the probability for STBC3 is the worst. However, the four STBCs can easily be classified.

**Fig. 3:**
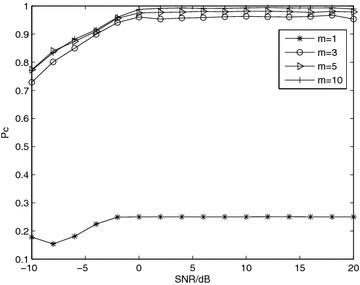
Average probability of correct classification, *Pc*, versus SNR with QPSK modulation and *N* = 8192 for diverse Nakagami-*m* fading channel

*Simulation 3* Influence of fading channel. Figure 3 shows the $P_c$ achieved with the proposed algorithm over Nakagami-*m* fading channel with *m* = 1, 3, 5, 10. As expected, the performance improves as *m* increases. For example, at *SNR* = 5 dB, $P_c=0.25,0.9685,0.9772,0.9915$ for *m* = 1, 3, 5, 10, respectively, while it reaches 1 at $+\infty$. This can be easily explained, as the variance of the channel coefficients increases for lower *m* values, which affects the value of the discriminating peaks, thus, leading to erroneous decisions.

**Fig. 4:**
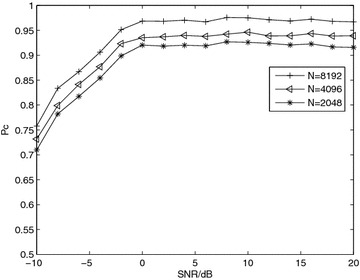
Average probability of correct classification, *Pc*, versus SNR with QPSK modulation and Nakagami-*m* fading channel, *m* = 3 for diverse *N*

*Simulation 4* Influence of received samples. Figure 4 shows the effect of the received samples on the $P_c$. The performance enhanced by increasing the number of samples *N*, as this results in an increase of the peak values and reduction of the effect of the noisy components. A large number of samples is required for accurate estimation of the discriminating feature, since the proposed algorithm depends on fourth-order cumulants.

**Fig. 5:**
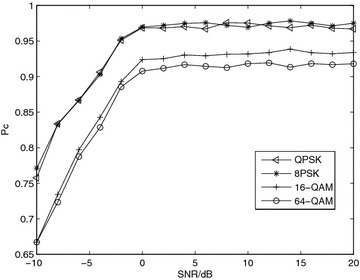
Average probability of correct classification, *Pc*, versus SNR with *N* = 8192 and Nakagami-*m* fading channel, *m* = 3 for diverse modulation

*Simulation 5* Influence of modulation. Figure 5 shows the effect of the modulation on the $P_c$. We have evaluated the behavior of our algorithm for 4 complex modulation: QPSK, 8PSK, 16-QAM and 64-QAM. These modulations are mandatory for most of the wireless standards. A better performance is showed for *M*-PSK signals when compared with *M*-QAM signals. The explanation is that the cumulants is constant for *M*-PSK, whereas it is not for *M*-QAM.

**Fig. 6:**
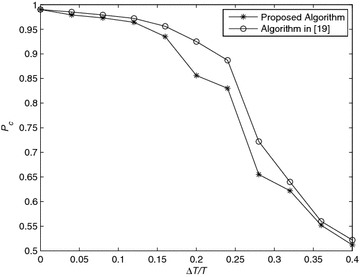
Average probability of correct classification, *Pc*, versus SNR with QPSK modulation and *N* = 8192 over Nakagami-*m* fading channel, *m* = 3 for timing offset

*Simulation 6* Influence of time offset. Figure 6 shows the effect of time offset and the performance of the algorithm in Eldemerdash et al. (2013a). The timing offset is normalized to the sampling period, $0\le \mu \le 1$. For the case of rectangular pulse shaping, after the matched filtering, the timing offset $\mu$ translates into a two path channel $[1-\mu , \mu ]$ (Swami and Sadler 2000). The performance of the proposed algorithm is more sensitivity to timing offsets than the algorithm in Eldemerdash et al. (2013a).

**Fig. 7:**
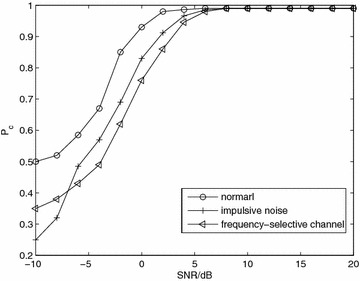
Average probability of correct classification, *Pc*, versus SNR with QPSK modulation and *N* = 8192 over Nakagami-*m* fading channel, *m* = 3 for impulsive noise and frequency-selective channels

*Simulation 7* Influence of impulsive noise and frequency-selective channels. Figure 7 shows the performance over impulsive noise and frequency-selective channels. The impulsive noise is characterized by a two-term Gaussian mixture given in Swami and Sadler (2000), and the frequency-selective channels is given in Marey et al. (2014). From the figure, we can see that the performance is worse with non-Gaussian noise and frequency-selective channels. When SNR ≥ 5 dB, the probability of correct classification is almost 1.

**Fig. 8:**
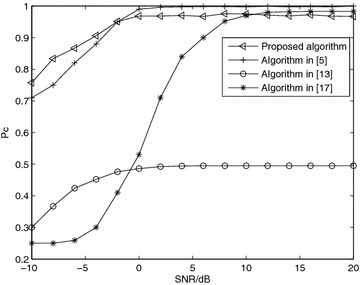
Average probability of correct classification, *Pc*, versus SNR with QPSK modulation and *N* = 8192 over Nakagami-*m* fading channel, *m* = 3 for different algorithms

*Simulation 8* Performance comparison. Figure 8 shows the comparison among the proposed algorithm, the optimal likelihood-based algorithm in Choqueuse et al. (2010), the second-order correlation-based algorithm in Choqueuse et al. (2008a), and the discrete Fourier transform (DFT)-based algorithm in Eldemerdash et al. (2013a).

The performance of the optimal likelihood-based algorithm is best, but it require estimation of the channel, noise information of the transmitted signal. The proposed algorithm does not need these estimation and more suitable to reality system. The performance of the proposed algorithm greatly outperforms the algorithm in Choqueuse et al. (2008a), which achieves a $P_c=0.5$ even for high SNR. This can be explained as the second-order correlation provides a discrimination feature for SM, ST3, and ST4 only. For Al, it equals zero, leading to the mis-classification. Algorithm (Eldemerdash et al. 2013a) has a little better performance than the proposed algorithm when SNR > 8 dB, but in low SNR, it is on the contrary. The probability of classification of proposed algorithm researches 0.97 at 0 dB, when algorithm (Eldemerdash et al. 2013a) at 8 dB.

## Complexity comparison

The complexity of the proposed algorithm is $O(N\log {N})$. which is the same with algorithms in Choqueuse et al. (2008a), Marey et al. (2014), Mohammadkarimi and Dobre (2014) and Eldemerdash et al. (2013a).

## Conclusion

This paper proposed an algorithm for blind classification of STBC using a single antenna based on high-order cumulants. We have shown that simple HOS are useful for classification of STBC. The algorithm was evaluated through simulations in terms of average probability of correct classification. The proposed algorithm, with the advantages that it does not require channel estimation and noise information, performed better than any other classification algorithms using a single antenna in low SNR. Moreover, it can benefit from spatially correlated fading.

The decision thresholds were on the conservative side because they were obtained by assuming that the sample estimates of the test statistics $C_{40}$ and $C_{42}$ have equal variances under different hypotheses, and ignored the effects of additive noise. The performance could be improved by taking these issues into account.

## Footnotes

1 We choose Al and SM as they are the most commonly used in wireless standards, and ST3 and ST4, as being commonly referred codes.

2 In this article, we do not consider $C_{41}$ (Swami and Sadler 2000).
